# Supplementary material for: A high-resolution mRNA expression time course of embryonic development in zebrafish
Source: eLife. 2017 Nov 16;6:e30860. doi: 10.7554/eLife.30860 (PMC5690287; doi:10.7554/eLife.30860)
Supplement: Supplementary file 6. [file elife-30860-supp6.zip › biolayout-clusters-files/Cluster148-genes.html]

Cluster148


# Cluster148: Genes

| Ensembl ID | Gene Name | Chr | Start | End | Biotype |
| --- | --- | --- | --- | --- | --- |
| ENSDARG00000032426 | ADCK3 (1 of many) | 17 | 6310168 | 6330946 | protein\_coding |
| ENSDARG00000103125 | TSC2 | 1 | 53523470 | 53594697 | protein\_coding |
| ENSDARG00000039343 | ahcyl2 | 4 | 5017055 | 5083765 | protein\_coding |
| ENSDARG00000006816 | gtf2f2b | 9 | 18821648 | 18897605 | protein\_coding |
| ENSDARG00000020529 | hacl1 | 16 | 20361992 | 20388566 | protein\_coding |
| ENSDARG00000011233 | pcyt1aa | 2 | 9957602 | 9982139 | protein\_coding |
| ENSDARG00000069324 | vps53 | 15 | 25165464 | 25218138 | protein\_coding |
| ENSDARG00000007614 | wwox | 25 | 36389835 | 36500147 | protein\_coding |
| ENSDARG00000075747 | zfyve16 | 10 | 2948797 | 2999352 | protein\_coding |
